# Immunogenicity evaluation of plasmids encoding *Brucella melitensis* Omp25 and Omp31 antigens in BALB/c mice

**DOI:** 10.22038/IJBMS.2018.27540.6722

**Published:** 2018-09

**Authors:** Moslem Shojaei, Mojtaba Tahmoorespur, Mahdi Soltani, Mohammad Hadi Sekhavati

**Affiliations:** 1 Department of Animal Science, Faculty of Agriculture, Ferdowsi University of Mashhad, Mashhad, Iran; 2 Department of Biotechnology, Institute of Science and High Technology and Environmental Sciences, Graduate University of Advanced Technology, Kerman, Iran

**Keywords:** Brucella melitensis, DNA vaccine, Omp25, Omp31, Protective immunity

## Abstract

**Objective(s):**

Vaccination is one of the most effective means to protect humans and animals against brucellosis. Live attenuated Brucella vaccines are considered effective in animals but they may be potentially infectious to humans, so it is vital to improve the immunoprotective effects and safety of vaccines against Brucella. This study was designed to evaluate the immunogenicity of DNA vaccines encoding *B. melitensis* outer membrane proteins (Omp25 and Omp31) against *B. melitensis* Rev1 in a mouse model.

**Materials and Methods:**

For this propose, Omp25 and Omp31 genes were cloned (individually and together) into the eukaryotic expression vector pcDNA3.1/Hygro (+). Expressions of recombinant plasmids were confirmed by SDS-PAGE and Western blot analysis. Six groups of BALB/c mice (seven mice per group) were intramuscularly injected with three recombinant constructs, native pcDNA3.1/Hygro (+) and phosphate-buffered saline (PBS) as controls and subcutaneous injection of attenuated live vaccine Rev1.

**Results:**

Results indicated that DNA vaccine immunized BALB/c mice had a dominant immunoglobulin G response and elicited a T-cell-proliferative response and induced significant levels of interferon gamma (INF-γ) compared to the control groups.

**Conclusion:**

Collectively, these finding suggested that the pcDNA3.1/Hygro DNA vaccines encoding Omp25 and Omp31 genes and divalent plasmid were able to induce both humoral and cellular immunity, and had the potential to be a vaccine candidate for prevention of *B. melitensis* infections.

## Introduction

Brucellosis is the most common zoonotic disease, with over half a million annual new human cases worldwide (1). Vaccination considered as the most effective tool to protect against infectious diseases (2), but there is no licensed vaccine for prevention of human brucellosis. Various vaccine modalities, including DNA, protein, viral vector, and live attenuated vaccines, have been developed for protection against brucellosis (3). Attenuated vaccine strains (like *Brucella melitensis* Rev1), although are effective, are less protective than ideal goal since they have disadvantages and can be unsafe and infectious for humans. DNA vaccine can incite both cellular and humoral immunity responses and provide longer antigen expression, that leading to more immune responses and induce memory responses against infectious agents (4).

Omp31 and Omp25 are major *Brucella* outer membrane proteins (Omps) (5). Omp31 protein is one of the major immunodominant proteins in *B. melitensis* as previously described by Bowden *et al.* (6). Antibodies against Omp31 have been detected in sheep naturally and experimentally infected with *B. melitensis* and have shown to protect mice against *B. melitensis* and *Brucella ovis* challenges (7). PCR–RFLP analysis of Omp25 suggested that the Omp25 gene is highly conserved in *Brucella* species, biovars and strains (8). DNA vaccine harbouring Omp25 of *B. melitensis* was protective against the virulent *B. melitensis* challenge in mice (9). Many reports have shown that Omp25 and Omp31 genes have particular usefulness for vaccination against sheep and goat brucellosis and for developing new generation vaccines against brucellosis (5, 10). Some evidences showed that multiple plasmid construct as DNA vaccines, can induce more immunization and effectively protection rather than monovalent constructs (11).

In this study, Omp25 and Omp31 were employed to design monovalent and divalent DNA vaccines and compare their potential abilities to protect against *Brucella* infection in a mouse model.

## Materials and Methods

***Animals***

Forty-two seven-to-eight-week-old female BALB/c mice (Neurosciences Research Center, Kerman, Iran) were acclimatized at least for one week before of injections and randomly distributed into experimental groups (six groups and seven mice per groups). Mice were kept in conventional animal facilities and received water and food *ad libitum* and were handled and disposed according to the guidelines of Ethical Committee for Animal experiments of the Kerman University of Medical Sciences (Iran).

***Bacterial strains and plasmids***

*Escherichia coli* DH5α was used as host strain. Live attenuated *B. melitensis* Rev1 strain was used for DNA template extraction and as control in mice immunization that obtained from Veterinary Organization Pharmacy of Kerman city. pTZ57R/T (T/A cloning vector) and pcDNA3.1/Hygro (expression vector) (Thermo Fisher Scientific, USA) were used for cloning, sequencing and preparation of DNA vaccines.

***Cloning into pTZ57R/T vector***

Total DNA was extracted from purified *B. melitensis* strain Rev1 using DNeasy Blood & Tissue Mini spin column DNA extraction kit (QIAGEN, Germany) according to the manufacturer’s instructions. Primers (O25 and O31) were designed using CLC Main Workbench 5.5 (CLC bio, Denmark) and Primer Premiere (Premier Biosoft, USA) softwares based on published Omp25 and Omp31 gene sequences deposited in the NCBI GenBank and were synthesized by Macrogen Co (South Korea). Amplification reactions were performed in a 25 μl volume using 1.5 unit of *pfu* DNA polymerase. For TA cloning, 3´-A overhangs added to fragments. Tailed PCR products were ligated into pTZ57R/T vector (Thermo Fisher Scientific, USA) based on TA cloning scheme according to the manufacturer’s instructions. Recombinant plasmids were transfered to competent *E. coli* DH5α cells (12). Transformed colonies were screened by ampicillin resistance (100 μg/ml) and blue-white selection. Success of recombination was confirmed by colony PCR and sequencing. The nucleotide sequences of genes were submitted to BLAST search at NCBI server (http://www.ncbi.nlm.nih.gov/blast/) to compare with sequences presented in the GenBank.

***Plasmid construct encoding Omp25 and Omp31 genes***

Based on obtained Omp25 and Omp31 sequences and considering the desired expression vector sequence, proper restriction enzymes were selected and inserted in designed primers for directional cloning of construct into expression vectors (Table I). Obtained PCR products contained desirable restriction sites were ligated into pcDNA3.1/Hygro vector and ligation product transformed into DH5α competent cells. Recombination confirmed by colony PCR, enzyme digestion of recombinant plasmids and comparison with native plasmids.

***Construction of divalent pcDNA3.1-Omp25-31 plasmid***

To construct the recombinant plasmid pcDNA3.1 with fusion gene (Omp25-31), proper restriction enzymes were selected and inserted in PCR primers and TAA stop codon was removed from Omp25 gene (Table I). The Full-length ORF of two Omp25 and Omp31 genes was amplified by Dpc25 and Dpc31 primers using PCR. After double digestion of Omp25 and Omp31 PCR products with BamHI/EcoRI and EcoRI/XhoI enzymes respectively, ligated together with T4 DNA ligase enzyme (Thermo scientific, USA). Divalent produced product was then ligated into BamHI/XhoI pre-digested pcDNA3.1/Hygro (+) vector. This process causes that the two genes inserted to vector together in one open reading frame. Recombination confirmed by colony PCR using forward primer of first gene (Dpc25-F) and reverse primer of second gene (Dpc31-R), enzyme digestion of recombinant plasmids and comparison with native plasmid.

***Eukaryotic expression of recombinant plasmids***

Hela cell line was cultured in complete RPMI 1640 medium (Gibco, USA) supplemented with 10 mM HEPES (Sigma, Germany), 1 mM 2ME (Sigma, Germany), 10% heat-inactivated foetal bovine serum (FBS-Gibco, USA), and penicillin–streptomycin (Gibco, USA). All plates were incubated in 5% CO_2_ incubator at 37 ^°^C, overnight. After incubating for 12 hr, these cells were transfected with 0.5 µg of recombinant plasmids using X-tremeGENE HP DNA (Roche, Switzerland) according to the manufacturer’s instructions. Post-transfection drug-resistant cells separated with culture in medium supplemented with 100 μg/ml hygromycin. After 48 hr, the cells were harvested for later analyses.

***SDS-PAGE and Western blot***

Transfected cells were harvested and washed once with 1X PBS. The cell pellets were resuspended in 200 µl lysis buffer (100 mM NaCl, 20 mM Tris-HCl, pH 7.5, 1 mM EDTA, 0.5% Triton X-100 and 1 mM PMSF). The lysates of Hela cells were applied in 12% SDS-PAGE gels and after staining with Coomassie brilliant blue (AppliChem, Germany) protein bands were visualised. Recombinant Opm25, Omp31 and Omp25-31 proteins were identified using Western blot by electrotransfer of proteins onto polyvinylidene difluoride filter (PVDF) membrane using an electrophoresis transfer system (Bio-Rad, USA) at 120 V for 2 hr and followed by antigen-antibody reactions. The blots were blocked in with Tris-buffered saline (TBS) (10 mM Tris-HCl, pH=8, 150 mM NaCl) plus 0.1% Tween 20 (TBS-T) containing 5% fat-free milk powder overnight at 4 ^°^C, and then incubated with 1:1000 dilution of mouse anti *B. melitensis* Rev1 serum (13) with TBS-T at room temperature for 2 hr. Blots were washed three times with TBS. Finally bounded antibodies were visualized with rabbit anti-mouse IgG horseradish peroxidase-conjugated secondary antibody (diluted 1:10000, Santa cruz, Biotechnology, USA) and developed by enhanced chemiluminescence assay (Amersham, UK) according to the manufacturer’s instructions.

***Immunization of BALB/c mice***

Plasmid DNA was isolated using GeneJET Plasmid Miniprep Kit (Thermo Fisher Scientific, USA) according to the manufacturer’s instructions. Mice were injected with 50 µg of recombinant plasmids diluted in 100 µl of PBS in each tibialis anterior muscle. Mice were vaccinated at days 0, 14, 21 and 28. Positive (injection of 2.2×10^4^ live attenuated *B. melitensis* Rev1) and negative control groups (injection of native pcDNA3.1 or PBS) were considered in immunization experiments. Sera samples were obtained after each immunization and spleens were removed after last immunization for immune response analysis.

***Induced humoral immune responses***

To evaluate the induced humoral immune response, after collection of mice immunized blood samples from their orbital sinuses at 0, 14, 28 and 42 days after injection, serum antibody titres were evaluated by indirect ELISA. Briefly, a 96-well plate was coated with lysate of cells carrying candidate DNA vaccine expressed recombinant Omp25 and OMP31 (100 µl/well). After overnight incubation, coated plate, blocked with 5% bovine serum albumin (BSA) solution. Washing step repeated once, and then incubated with the mice serum in 1:100 dilution at 37 ^°^C for 3 hr. Subsequently, serum antibody titres were determined using rabbit anti-mouse IgG–HRP conjugated antibodies (Santa Cruz Biotechnology, USA) and absorbance were recorded at 490 nm (OD_490_) after 30 min incubation. 

***Lymphocyte proliferation and MTT assay***

Two weeks after the last immunization, five mice per group were sacrificed, and their spleens were removed under aseptic conditions and were homogenized in 2 ml of tissue culture medium (RPMI 1640 with 5% FBS). After centrifugation, collected cells resuspended in 4 ml 0.75% Tris–NH4Cl (pH 7.4) to lyse erythrocytes. After washing three times with PBS, the splenocytes were resuspended in RPMI 1640 medium (Gibco, USA), supplemented with 10% FBS (Gibco, USA), and 2 mM l-glutamine. Splenocytes were seeded in 96-well plate (100 μl per well = 4×10^6^ cells per well) in triplicate. Splenocytes of each immunized group in each well were stimulated with *B. melitensis* Rev1 bacterial lysate (10 µg/ml), concanavalin A as a positive mitogen (Con A, 3 µg/ml; Sigma, USA) and no additives (unstimulated control). One hundred µl of complete RPMI-1640 medium were used in blank control wells. After 72 hr of incubation at 37 ^°^C in 5% CO_2_, 20 µl of MTS (Promega, USA) was added to each well. The absorbance of each well was measured at 570 nm (OD_570_) by ELISA reader (Bio-Tek, USA) after incubated for 4 hr. Finally, for each experimental groups the stimulation index (SI) was calculated as the ratio of differences of the absorbance values of stimulated cells with antigen and Con A of blank RPMI wells to the unstimulated cells using the below formula (14):

SI=mean OD of stimulated culture-mean OD of RPMI blank mean OD of unstimulated culture-mean OD of RPMI blank

***Cytokine detection***

Levels of interferon-gamma (IFN-ɣ) and interleukin-10 (IL-10) cytokines in supernatants of splenocyte culture of immunized mice were measured after 72 hr of incubation with antigens. For these purpose, spleen cells (approximately 4×10^6^ cells/ml) from pcDNA3.1-Omp25, PcDNA3.1-Omp31, PcDNA3. 1-Omp25-31 and live attenuated Rev1 immunized mice were stimulated with lysate of *B. melitensis* Rev1 bacteria (10 µg/ml) and 3 µg/ml concanavalin A (as positive control) for 72 hr, and complete RPMI 1640 medium used as blank. The supernatant of immunized mice with native pcDNA3.1 plasmid and PBS were used as controls. IFN-ɣ and IL-10 in culture supernatants were measured by sandwich ELISA using paired cytokine specific monoclonal antibodies (mAbs) according to the manufacturer’s instructions (Pharmingen, CA).

**Table 1:** PCR primers for construction of monovalent and divalent plasmid constructs

**Gene**	**Primer**	**Primer sequence** *	**Enzyme**	**Ta** ٭٭
Omp25	O25-F O25-R	**ATG**CGCACTCTTAAGTCTCTCGT **TTA**GAACTTGTAACCGATGCCGACG	­	58 ºC
Omp31	O31-F O31-R	**ATG**AAGTCCGTAATTTTGGCGTCC **TTA**GAACTTGTAGTTCAGACCGACG	­	55 ºC
Omp25	Pc25-F Pc25-R	GGGGTACCGCCACC**ATG**CGCACTCTTAAGTCTC CGCGGATCCGG**TTA**GAACTTGTAGCCGATGCCG	*Kpn*I *Bam*HI	60 ºC
Omp31	Pc31-F Pc31-R	CCCAAGCTTGCCACC**ATG**AAGTCCGTAATTTTGGC CGCGGATCCGGCAA**TTA**GAACTTGTAGTTCAGACCG	*Hind*III *Bam*HI	57 ºC
Omp25	Dpc25-F Dpc25-R	CGCGGATCCGCCACC**ATG**CGCACTCTTAAGTCTC CCGGAATTCGAACTTGTAGCCGATGCCGACGCGG	*Bam*HI *Eco*RI	61 ºC
Omp31	Dpc31-F Dpc31-R	CCGGAATTC**ATG**AAGTCCGTAATTTTGGCGTCC CCGCTCGAG**TTA**GAACTTGTAGTTCAGACCGAC	*Eco*RI *Xho*I	56 ºC

٭ Restriction sites are underlined, and initiation and termination codons are shown in boldface

** The thermal profile for all reactions was as follows: 95 ºC: 5 min; 35X (95 ºC: 45 sec, Ta: 45 sec, 72 ºC: 45 sec); 72 ºC: 5 min.

**Figure 1:**
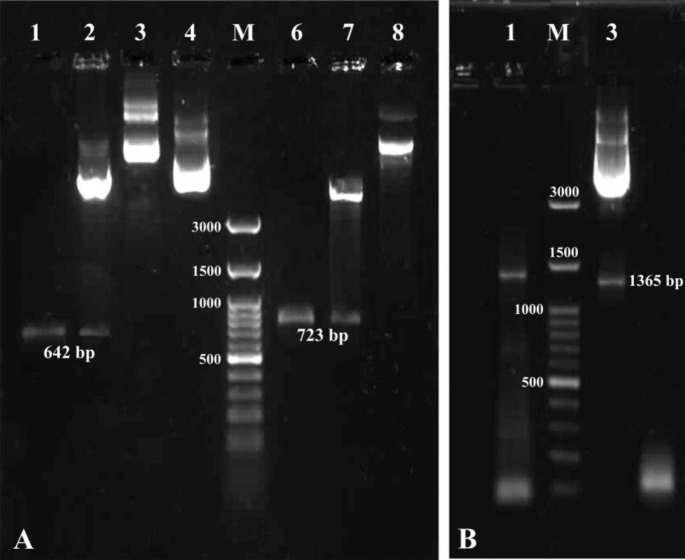
Recombinant confirmation of constructs. (**A)**: lane1, amplification of *B. melitensis* Rev1 Omp25 gene; lane 2, double enzyme digestion (*Kpn*I/*Bam*HI) of pcDNA3.1-Omp25 recombinant construct; lane 3, recombinant pcDNA3.1/Hygro-Omp25; lane 4, native pcDNA3.1/Hygro (+); lane M, 100 bp DNA ladder (Fermentas); lane 6, amplification of *B. melitensis* Rev1 Omp31 gene; lane 7, double enzyme digestion (*Hind*III/*Bam*HI) of pcDNA3.1-Omp31 recombinant construct, lane 8, recombinant pcDNA3.1/Hygro-Omp31. (**B)**: lane 1, colony PCR of Omp25-31 fragment; lane 2, 100 bp DNA Ladder (Fermentas); lane 3, double enzyme digestion (*Bam*HI/*Xho*I) of divalent pcDNA3.1-Omp25-31 recombinant construct.

**Figure 2:**
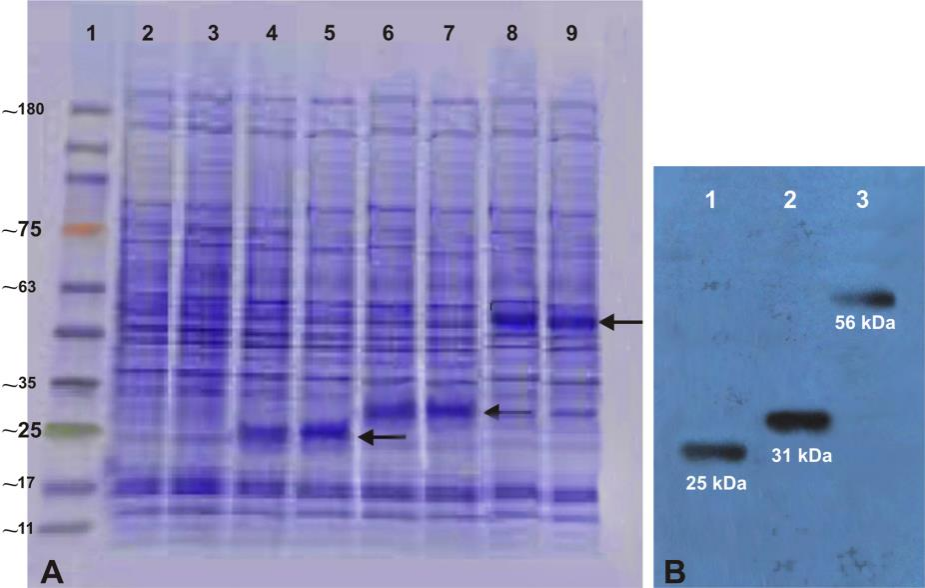
Expression and analysis of recombinant plasmids in Hela cells. The expression of recombinant plasmids were screened by SDS-PAGE, (**A**); lane 1 Tri-Color Prestained Protein MW Marker (EZ BioResearch, USA); lane 2 and 3 lysate of Hela cells without transformation and lane 4 and 5 lysate of Hela cells transformed with the recombinant pcDNA3.1/Hygro-Omp25, lane 6 and 7 lysate of Hela cells transformed with recombinant pcDNA3.1/Hygro-Omp31 and lane 8 and 9 lysate of Hela cells transformed with divalent pcDNA3.1/Hygro-Omp25-31 plasmids and western blot assays (**B**); lane 1, recombinant Omp25 protein (~25 kDa), lane 2, recombinant Omp31 protein (~31 kDa) and lane 3, recombinant Omp25-Omp31 protein (~56 kDa) detected of Hela cells proteins transferred on PVDF membrane.

**Figure 3:**
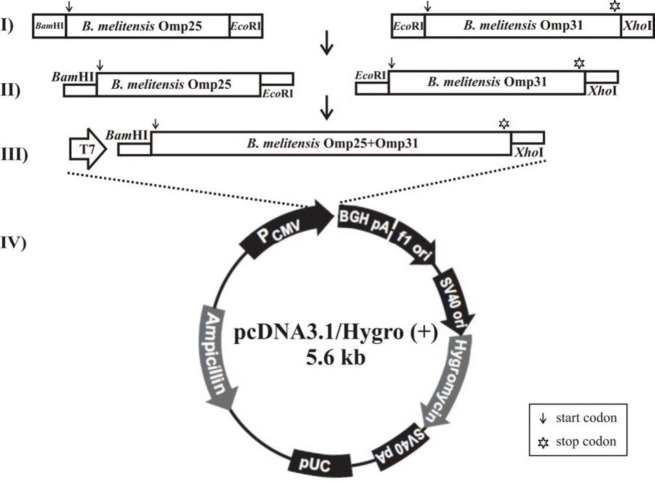
Schematic showing of construction of divalent recombinant pcDNA3.1/Hygro-Omp25-Omp31, I) Amplification of *B. melitensis* Omp25 and Omp31 genes using Table I primers by PCR. II) Double digestion of PCR products, III) Ligation of digested product by T4 Ligase, IV) Insertion of divalent Omp25-Omp31 fragment into digested pcDNA3.1/Hygro (+).

**Figure 4:**
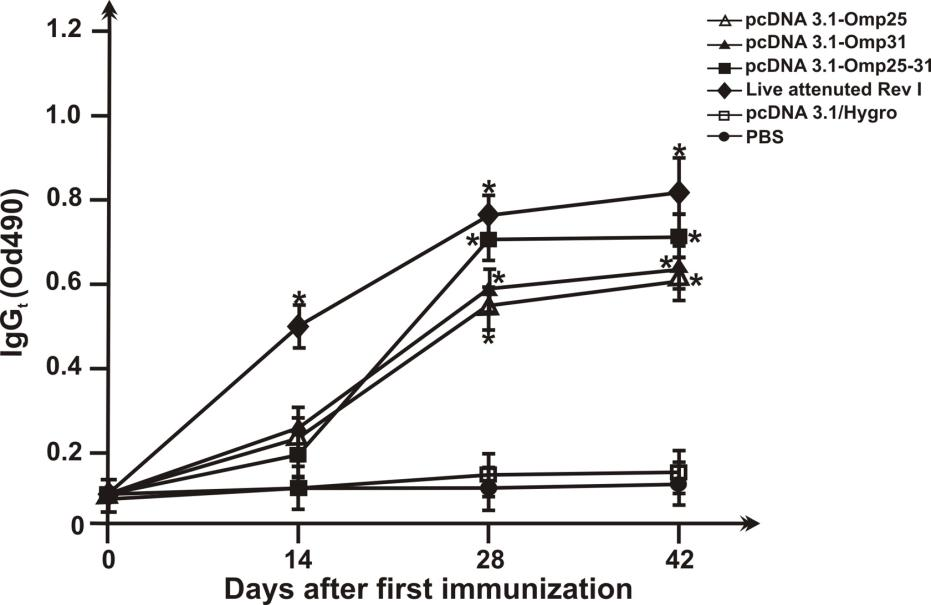
Serum IgG titers of mice immunized. Sera were collected from the seven groups of immunized BALB/c mice with different DNA vaccines (pcDNA3.1/Hygro-Omp25, pcDNA3.1/Hygro-Omp31 and pcDNA3.1/Hygro-Omp25-31) and native pcDNA3.1/Hygro and PBS and IgG titers were evaluated by a quantitative ELISA. Each value represents the mean OD ± standard deviation of antibodies. The statistical significance is represented by *P*< 0.001 compared with the control native pcDNA3.1 and PBS- immunized groups

**Figure 5:**
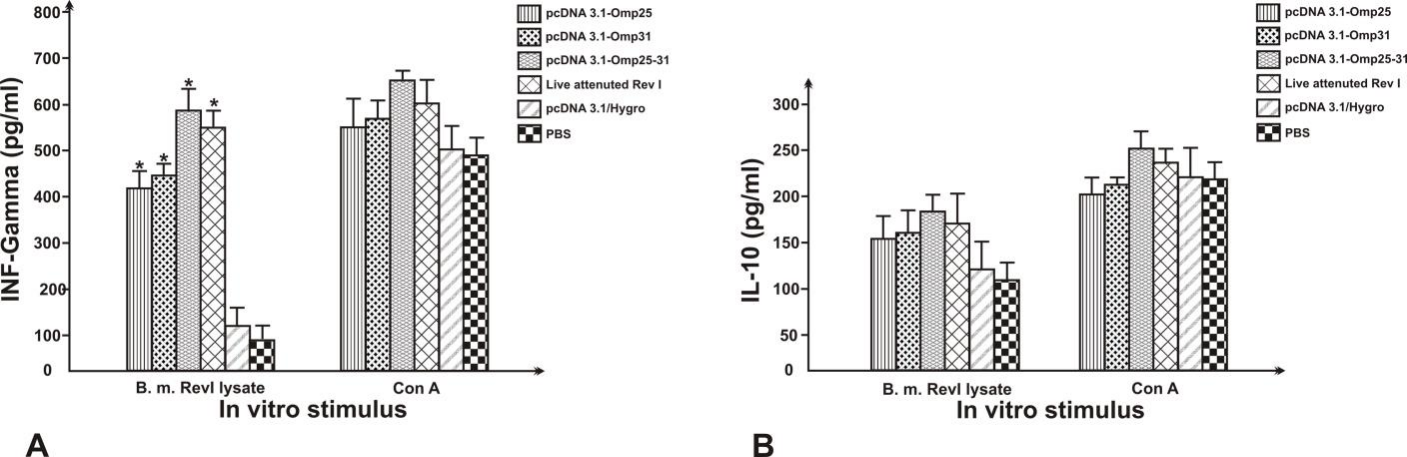
ELISA analysis of IFN-ɣ (A) and IL-10 (B) from BALB/c mice inoculated with DNA vaccines (pcDNA3.1/Hygro-Omp25, pcDNA3.1/Hygro-Omp31 and pcDNA3.1/Hygro-Omp25-31), attenuated live vaccine, Phosphate buffer Saline and native plasmid as negative controls. IFN-ɣ and IL-10 in cell supernatants were quantified (pg/ml) by ELISA. Each value represents the mean ± S.D. of the response of spleen cells from each group. Data are representative of two separate experiments. “*”, significantly different from mice immunized with PBS (P< 0.01).

**Figure 6:**
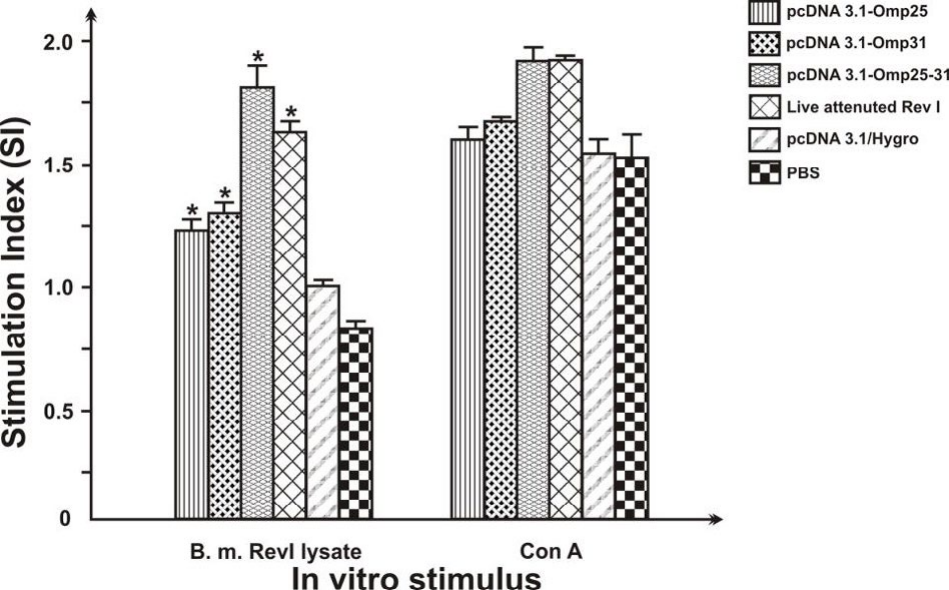
Lymphocyte proliferation assay responses of spleen cells from immunized BALB/c mice with pcDNA3.1-Omp25-31, pcDNA3.1-Omp25, and pcDNA3.1-Omp31 DNA vaccines, native pcDNA3.1/Hygro, PBS, and Rev1 vaccine (five mice per group). Mice immunized with PBS were used as controls. Spleen cells from immunized mice were stimulated *in vitro* with Rev1 bacteria lysate and Con A*. *SI values show the lymphocyte proliferative activity for each group that were calculated as the ratio between the difference of mean absorption values at 570 nm of stimulated group (with antigen and Con A) of blank RPMI to the difference of unstimulated groups. Data represent the mean SI ± standard deviation from each six group. “*” significantly different compared with mice immunized with PBS (*P*< 0.01).

**Statistical analysis**

Immune response data were analysed using t-test between two groups and ANOVA analysis among several groups. Statistical significance was assumed at the *P*<0.01 level. The statistical analyses were performed using the GraphPad Prism 5 software.

## Results

***Amplification, cloning and sequencing of DNA coding for Omp25 and Omp31***

A-tailed PCR products of open reading frames of Omp25 and Omp31genes were successfully ligated into pTZ57R/T vector by TA cloning scheme. Selected white colonies generated strong bands after colony PCR that showed recombination process was done as expected. The nucleotide sequence of Omp25 and Omp31 genes were composed of 642 and 723 bp respectively that revealed very high similarity with other *B. melitensis* recorded sequences and submitted in the GenBank database (NCBI) under the accession numbers KY021737 and KX100580, respectively.

***Construction and expression of monovalent DNA vaccines***

Amplified Omp25 and Omp31 gene fragments were successfully cloned into pcDNA3.1/Hygro (+) and recombinant mammalian expression constructs were built. Success of recombination processes were confirmed by colony PCR, comparison with native pcDNA3.1/Hygro and digestion with restriction enzymes *Kpn*I/*Bam*HI and *Hind*III/*Bam*HI (Figure 1). SDS-PAGE analyses of the lysate of transfected Hela cells carrying

the DNA vaccines showed the expected recombinant protein pattern with the molecular mass of approximately 25 and 31 kDa for pcDNA3.1-Omp25 and pcDNA3.1-Omp31, respectively. Western blot procedure resulted in dark violet colour band at a location corresponding to ~25 kDa and ~31 kDa that confirmed the expression of Omp25 and Omp31 proteins in the eukaryotic system (Figure 2).

***Construction and expression of divalent DNA vaccine***

Omp25 and Omp31 gene fragments were successfully amplified and ligated. Resulting construct consisted of two genes in one open reading frame with *Bam*HI/*Xho*I restriction sites. Digested ligated product (Omp25-Omp31) was correctly inserted into digested pcDNA3.1/Hygro (Figure 3). Construction of recombinant divalent plasmid confirmed by colony PCR, enzyme digestion and sequencing (Figure 1). As shown in Figure 2, the presence of Omp25Omp31 protein was detected by SDS-PAGE analysis and Western blotting of cell lysates of Hela cells transfected with the divalent pcDNA3.1-Omp25-31 plasmid confirmed the ability of pcDNA3.1-Omp25-31 to produce the chimeric protein Omp25-Omp31 in eukaryotic Hela cells.

***Humoral immune responses***

As shown in Figure 4, immunization with recombinant plasmid constructs elicited noticeable IgG responses that the titres of IgG began to increase from the first week of injection until the sixth week after immunization, and peaked at the sixth week after immunization. The serum IgG antibody levels in the pcDNA3.1-Omp25-31 and attenuated live vaccine groups were significantly higher than those in the monovalent DNA vaccine and control groups (*P*< 0.01). The serum IgG antibody levels in the pcDNA3.1-Omp31 were slightly higher than that in pcDNA3.1-Omp25 group although the increases were not significant. The groups inoculated with native pcDNA3.1/Hygro and PBS did not produce any significant anti-fusion antibodies.

***Cellular immune response***

Whereas cytokines produced by activated T cells are indicators of the type of Th responses, IFN-ɣ and IL-10 secretion by the re-stimulated splenocytes was evaluated by ELISA. DNA-immunized group induced higher levels of IFN-ɣ in comparison to PBS and native plasmid-immunized group (P< 0.01). B. melitensis Rev1 immunized group also induced higher levels of IFN-ɣ in comparison to PBS-immunized group (*P*< 0.01). The significant cytokine production was not detected from PBS and pcDNA3.1/Hygro immunized mice. There was no statistically significant difference between the levels of the IL-10 in vaccine groups and negative control groups (*P*> 0.01) (Figure 5). As shown in Figure 6, SI value obtained of Lymphocyte proliferation assay of pcDNA3.1-Omp25-31 construct immunized group induced more proliferative response to either the other plasmids or live attenuated Rev1. Also the result of cellular immune responses of mice immunization with PBS in control group shown to have any effect on the level of T-cell proliferative response. The SI values for mice immunized with pcDNA3.1-Omp25 DNA vaccine showed a similar proliferative response compared to pcDNA3.1-Omp31.

## Discussion

Brucellosis is a zoonosis disease with no effective available vaccine that is caused by facultative intracellular *Brucella* spp. bacteria. Both humoral and cellular immune responses have important roles for elimination of intracellular pathogens (15). Immunization with DNA plasmids encoding the immunogenic antigens that can induce both humoral and cellular immune responses represents an effective method in vaccine research to prevent this disease. In this study, an attempt was done to evaluate the immunogenicity of pcDNA3.1-Omp25 and Omp31 DNA vaccines and divalent construction of these genes (pcDNA3.1-Omp25-31) to elicit an immune response and protective immunity in BALB/c mice. The efficiency of DNA vaccination against a pathogen can be affected by the choice of antigen and insertion of multiple antigens. As transmembrane proteins spanning the outer cell membrane and peptidoglycan, Omp25 and Omp31 genes may be necessary for *Brucella* to cause disease in the ruminant host via attachment or invasion of the organism to the host cell. *Brucella* lacking these genes might fail to generate a humoral and cell-mediated immune response in infected animals, resulting in an exacerbation of disease (16).

Many studies have reported that protective immune responses against *Brucella* are associated with high levels of Th1 cytokines and IgG responses (14, 17). Intramuscular inoculation of the divalent DNA vaccine (pcDNA3.1/Hygro-Omp25-31) and their monovalent constructs (pcDNA3.1-Omp25 and pcDNA3.1-Omp31) could induce considerable levels of IgG antibody. For as much as, *Brucella *species induce a Th1 cytokine bias of immune response (18), in current experiment, antigen-specific production of Th1 cytokines (IFN-ɣ) and Th2 cytokines (IL-10) in response to antigen stimulating of immunized mice spleen cells, were observed. The results showed that these DNA constructs could produce remarkably IFN-ɣ cytokine T cells, and whereas IFN-ɣ is mainly induced by Th1 and CD8+ T lymphocyte cells, thereby indicating that these constructs can produce a principally Th1-type T-cell immune response. IFN-ɣ is an essential effector cytokine that stimulates the macrophages for efficient killing and clearance of intracellular *Brucella* in susceptible BALB/c mice (19). Vitry *et al.* (2012) have suggested that INF-γ producing CD4+ T cells have a major role in clearing the *Brucella* bacteria and CD8+ T cells and humoral responses have modest role to play (20). Findings of present study clearly showed that all three types of Omp25, Omp31 and pcDNA3.1based divalent vaccines were able to produce a significant increase in IFN-γ in all vaccinated mice when compared with control mice inoculated with intact vector. However, the levels of IL-10 T-cell cytokine had differences in experimental immunized mice groups compare with control groups (PBS and pcDNA3.1/Hygro) that had a slightly increase, but they were not significant. (Figure 5). These observations suggested that these recombinant DNA vaccines induced a Type one (Th1) immune response after immunization. These finding is similar to the result of other studies by Velikovsky *et al.* (2002), Sislema-Egas *et al.* (2012) and Ghasemi *et al.* (2015), were indicated the importance of antibody and Th1 immune responses in infections of *B. melitensis* in BALB/c mice (21, 22, 17).

For further investigation of the cellular immune response induced by DNA vaccines, the proliferation of lymphocytes two weeks after last vaccination was detected. The SI values for the three DNA vaccine constructs groups and attenuated live vaccine group were consistently higher than those for the negative control groups (*P*< 0.01). However, there is no difference in the values among the groups of attenuated live vaccine and divalent DNA vaccine (*P*>0.05). As indicated in Figure. 5 and 6, the spleens of mice vaccinated with divalent recombinant plasmid had stronger lymphocyte proliferative response and higher level of IFN-γ than other groups. Intramuscular injection (IM) pathway is the most common route of DNA administration. In this study, it was also found that IM injection of a DNA vector containing the DNA insert of *Brucella* Omp25 and Omp31 was able to generate a protection in equal levels compared to that observed in positive control mice vaccinated with subcutaneous injection of live vaccine Rev1. It is noticeable that both humoral immune responses were low at primary vaccination but increased to high levels post-primary vaccination that the result of various reported studies such as Ghasemi *et al.* (2015) (17) and Yin *et al.* (2016) (23) confirmed these results. Whereas, the induced responses with pCIp39 and pCIsp41 DNA vaccines constructed by Al-Mariri *et al.* (2014) were comparable to that induced by live Rev1 vaccine against *Brucella* 4 weeks after infection but it was less in 8 weeks post-infection (24). Thus it seems that a longer experimental time for the more investigation of the effect of DNA plasmids could be more suitable. Also it is considerable that some studies showing that a DNA vaccine encoding the Omp31, concerning to elicit immune responses against *Brucella* in mice, but induced a very weak humoral response (25). In contrast, Leclerq *et al.* (2002) demonstrated that they could induce a Th1 type of immune response using DNA vaccines expressing the *Brucella* groEL heat-shock protein without inducing any protective immune response against a challenge with *Brucella abortus* (26). They were only able to induce CMI response using the mammalian expression vector pCMV-link, whereas they were not very successful in expressing the GroEL protein using a designed mammalian expression vector pCMV-tPA to secrete an immunogen protein antigen.

Due to the more cell epitopes in multivalent vaccines than in monovalent forms, with construction of multivalent DNA vaccines it might be able to stimulate immunity against a range of pathogens (14). Moreover, because of the low antigen expression or lack of immune recognition, the use of monovalent constructions of DNA plasmids does not induce stronger immune responses than multivalent constructions. Thus in the present study, a divalent DNA vaccine (pcDNA3.1-Omp25-Omp31) was created by using the complete ORF of *B. melitensis* Omp25 and Omp31 genes and the changes in the levels of specific immune responses was compared to monovalent forms and live *Brucella* vaccines. As shown in results, divalent DNA vaccine could induce a higher degree of humoral and immune responses than controls and other experimental groups. Previously described divalent DNA vaccines against *Brucella *such as pcDNA3.1-L7/L12-Omp16 (27), pCIBLSOmp31 (28) and pcDNA3-Omp31-eae (29) that elicited an immune response against *E. coli* and *B. melitensis* infections, also revealed that divalent DNA vaccines could induce a more intensive humoral and immune response compared to monovalent and control live Rev1 vaccines.

## Conclusion

The results from a relatively limited number of studies and literatures indicate that the DNA vaccines are suitable for the induction of protective immunity and development of an effective and safe immunization strategy against intracellular pathogens such as *Brucella *species*.* Results of the current study indicate that pcDNA3.1 based DNA vaccine encoding the ORF of *B. melitensis* Omp25 and Opm31 genes could be useful candidates for the development of vaccines against brucellosis since they elicit humoral, Th and CTL responses against *Brucella*. As DNA vaccine construct encoding both Omp25 and Omp31 genes of *B. melitensis* (pcDNA3.1-Omp25-31) elicited strong and protective cellular and humoral immune responses against *Brucella* infection as similar as the best vaccine available (Rev1) in mice model, this double DNA vaccine construct could be a useful candidate for the development of more efficacious DNA vaccines against *B. melitensis* infections. Although many studies have been explored the value of nucleic acid vaccines against brucellosis that provided various levels of specific cellular immune responses in mouse model, however, it becomes obvious that there is need for further studies using several such genes encoding protective antigens simultaneously. Future studies need to be focused on selecting additional *Brucella* proteins or peptides that could be construct a multivalent vaccine against any species of *Brucella, *especially in animal.

## References

[1] Pappas G, Papadimitriou P, Akritidis N, Christou L, Tsianos E (2006). The new global map of human brucellosis. Lancet Infect Dis.

[2] Hilleman MR (2000). Vaccines in historic evolution and perspective: a narrative of vaccine discoveries. Vaccine.

[3] Yang X, Skyberg JA, Cao L, Clapp B, Thornburg T, Pascual DW (2013). Progress in Brucella vaccine development. Front Biol.

[4] Kutzler MA, Weiner DB (2008). DNA vaccines: ready for prime time?. Nature Rev Genet.

[5] Caro-Hernández P, Fernández-Lago L, de Miguel M-J, Martín-Martín AI, Cloeckaert A, Grilló M-J (2007). Role of the Omp25/Omp31 family in outer membrane properties and virulence of Brucella ovis. Infect Immun.

[6] Bowden RA, Cloeckaert A, Zygmunt MS, Bernard S, Dubray G (1995). Surface exposure of outer membrane protein and lipopolysaccharide epitopes in Brucella species studied by enzyme-linked immunosorbent assay and flow cytometry. Infect Immun.

[7] Zygmunt MS, Debbarh HS-A, Cloeckaert A, Dubray G (1994). Antibody response to Brucella melitensis outer membrane antigens in naturally infected and Rev1 vaccinated sheep. Vet Microbiol.

[8] Cloeckaert A, Verger J-M, Grayon M, Grepinet O (1995). Restriction site polymorphism of the genes encoding the major 25 kDa and 36 kDa outer-membrane proteins of Brucella. Microbiology.

[9] Commander NJ, Spencer SA, Wren BW, MacMillan AP (2007). The identification of two protective DNA vaccines from a panel of five plasmid constructs encoding Brucella melitensis 16M genes. Vaccine.

[10] Vahedi F, Talebi A, Ghorbani E, Behroozikhah A, Shahriari Ahmadi F, Mahmoudi M (2011). Isolation, cloning and expression of the Brucella melitensis Omp31 gene. Iran J Vet Res.

[11] Johnston D, Bystryn J-C (2005). Heterogeneous antibody response to polyvalent melanoma vaccines in syngeneic mice. Cancer Immunol Immunother.

[12] Sambrook J, Fritsch E, Maniatis T (1989). Molecular Cloning A Laboratory Manual.

[13] Zheng W, Wang Y, Zhang Z, Yan F (2015). Immunological characteristics of outer membrane protein omp31 of goat Brucella and its monoclonal antibody. Genet Mol Res.

[14] Yang Y, Wang L, Yin J, Wang X, Cheng S, Lang X (2011). Immunoproteomic analysis of Brucella melitensis and identification of a new immunogenic candidate protein for the development of brucellosis subunit vaccine. Mol Immunol.

[15] Barquero-Calvo E, Martirosyan A, Ordoñez-Rueda D, Arce-Gorvel V, Alfaro-Alarcón A, Lepidi H (2013). Neutrophils exert a suppressive effect on Th1 responses to intracellular pathogen Brucella abortus. PLoS Pathog.

[16] Edmonds MD, Cloeckaert A, Booth NJ, Fulton WT, Hagius SD, Walker JV (2001). Attenuation of a Brucella abortus mutant lacking a major 25 kDa outer membrane protein in cattle. Am J Vet Res.

[17] Ghasemi A, Jeddi-Tehrani M, Mautner J, Salari MH, Zarnani A-H (2015). Simultaneous immunization of mice with Omp31 and TF provides protection against Brucellamelitensis infection. Vaccine.

[18] Denoel P, Vo T-O, Weynants V, Tibor A, Gilson D, Zygmunt M (1997). Identification of the major T-cell antigens present in the Brucella melitensis B115 protein preparation, Brucellergene OCB. J Med Microbiol.

[19] Murphy EA, Sathiyaseelan J, Parent MA, Zou B, Baldwin CL (2001). Interferon-γ is crucial for surviving a Brucella abortus infection in both resistant C57BL/6 and susceptible BALB/c mice. Immunology.

[20] Vitry M-A, De Trez C, Goriely S, Dumoutier L, Akira S, Ryffel B (2012). Crucial role of gamma interferon-producing CD4+ Th1 cells but dispensable function of CD8+ T cell, B cell, Th2, and Th17 responses in the control of Brucella melitensis infection in mice. Infect Immun.

[21] Velikovsky CA, Cassataro J, Giambartolomei GH, Goldbaum FA, Estein S, Bowden RA (2002). A DNA vaccine encoding lumazine synthase from Brucella abortus induces protective immunity in BALB/c mice. Infect Immun.

[22] Sislema-Egas F, Céspedes S, Fernández P, Retamal-Díaz A, Sáez D, Oñate A (2012). Evaluation of protective effect of DNA vaccines encoding the BAB1_0263 and BAB1_0278 open reading frames of Brucella abortus in BALB/c mice. Vaccine.

[23] Yin D, Li L, Song D, Liu Y, Ju W, Song X (2016). A novel recombinant multi-epitope protein against Brucella melitensis infection. Immunol Lett.

[24] Al-Mariri A, Akel R, Abbady A (2014). A DNA vaccine encoding p39 and sp41 of Brucella melitensis induces protective immunity in BALB/c mice. Arch Med Vet.

[25] Cassataro J, Estein SM, Pasquevich KA, Velikovsky CA, de la Barrera S, Bowden R (2005). Vaccination with the recombinant Brucella outer membrane protein 31 or a derived 27-amino-acid synthetic peptide elicits a CD4+ T helper 1 response that protects against Brucella melitensis infection. Infect Immun.

[26] Leclerq S, Harms JS, Rosinha GM, Azevedo V, Oliveira SC (2002). Induction of a Th1-type of immune response but not protective immunity by intramuscular DNA immunisation with Brucella abortus GroEL heat-shock gene. J Med Microbiol.

[27] Luo D, Ni B, Li P, Shi W, Zhang S, Han Y (2006). Protective immunity elicited by a divalent DNA vaccine encoding both the L7/L12 and Omp16 genes of Brucella abortus in BALB/c mice. Infect Immun.

[28] Cassataro J, Pasquevich KA, Estein SM, Laplagne DA, Velikovsky CA, de la Barrera S (2007). A recombinant subunit vaccine based on the insertion of 27 amino acids from Omp31 to the N-terminus of BLS induced a similar degree of protection against B ovis than Rev 1 vaccination. Vaccine.

[29] Ranjbar R, Sharifimoghadam S, Saeidi E, Jonaidi N, Sheikhshahrokh A (2016). Induction of protective immune responses in mice by double DNA vaccine encoding of Brucella melitensis Omp31 and Escherichia coli Eae genes. Trop J Pharma Res.

